# The Factors Associated With Nonuse of and Dissatisfaction With the National Patient Portal in Finland in the Era of COVID-19: Population-Based Cross-sectional Survey

**DOI:** 10.2196/37500

**Published:** 2022-04-22

**Authors:** Emma Kainiemi, Tuulikki Vehko, Maiju Kyytsönen, Iiris Hörhammer, Sari Kujala, Vesa Jormanainen, Tarja Heponiemi

**Affiliations:** 1 Finnish Institute for Health and Welfare Helsinki Finland; 2 Department of Industrial Engineering and Management Aalto University Espoo Finland; 3 Department of Computer Science Aalto University Espoo Finland

**Keywords:** patient portal, digital technology, eHealth, nonuse, dissatisfaction, COVID-19, digital health, patient empowerment, epidemiology, population survey, national survey

## Abstract

**Background:**

In the abnormal circumstances caused by the COVID-19 pandemic, patient portals have supported patient empowerment and engagement by providing patients with access to their health care documents and medical information. However, the potential benefits of patient portals cannot be utilized unless the patients accept and use the services. Disparities in the use of patient portals may exacerbate the already existing inequalities in health care access and health outcomes, possibly increasing the digital inequality in societies.

**Objective:**

The aim of this study is to examine the factors associated with nonuse of and dissatisfaction with the Finnish nationwide patient portal My Kanta Pages among the users of health care services during the COVID-19 outbreak. Several factors related to sociodemographic characteristics, health, and the use of health care services; experiences of guidance concerning electronic services; and digital skills and attitudes were evaluated.

**Methods:**

A national population survey was sent using stratified sampling to 13,200 Finnish residents who had reached the age of 20 years. Data were collected from September 2020 to February 2021 during the COVID-19 pandemic. Respondents who had used health care services and the internet for transactions or for searching for information in the past 12 months were included in the analyses. Bivariate logistic regression analyses were used to examine the adjusted associations of respondent characteristics with the nonuse of My Kanta Pages and dissatisfaction with the service. The inverse probability weighting (IPW) method was applied in all statistical analyses to correct for bias.

**Results:**

In total, 3919 (64.9%) of 6034 respondents were included in the study. Most respondents (3330/3919, 85.0%) used My Kanta Pages, and 2841 (85.3%) of them were satisfied. Nonusers (589/3919, 15%) were a minority among all respondents, and only 489 (14.7%) of the 3330 users were dissatisfied with the service. Especially patients without a long-term illness (odds ratio [OR] 2.14, 95% CI 1.48-3.10), those who were not referred to electronic health care services by a professional (OR 2.51, 95% CI 1.70-3.71), and those in need of guidance using online social and health care services (OR 2.26, 95% CI 1.41-3.65) were more likely nonusers of the patient portal. Perceptions of poor health (OR 2.10, 95% CI 1.51-2.93) and security concerns (OR 1.87, 95% CI 1.33-2.62) were associated with dissatisfaction with the service.

**Conclusions:**

Patients without long-term illnesses, those not referred to electronic health care services, and those in need of guidance on the use of online social and health care services seemed to be more likely nonusers of the Finnish nationwide patient portal. Moreover, poor health and security concerns appeared to be associated with dissatisfaction with the service. Interventions to promote referral to electronic health care services by professionals are needed. Attention should be targeted to information security of the service and promotion of the public’s confidence in the protection of their confidential data.

## Introduction

The worldwide COVID-19 pandemic limited the provision of nonurgent health care services [1,2]. During this time, the use and interest in patient portals increased [3,4] because portals have enabled patients to have continuous [5] and secure access to their health care documents and medical information [4]. Patient portals are electronic services that allow patients to access [6] and in some cases manage their electronic health record documentations [7] and interact with health care professionals [6,8,9]. The functionalities provided in a patient portal vary by portal and country [9,10].

Patient portals offer transparent information about the patients’ health and well-being [11] and enhance the delivery of individualized care [3]. Patient portals have been reported to increase the patients’ knowledge and understanding of their own health condition, and thus they might be better prepared for future contacts with health care professionals [12]. This supports patient empowerment and engagement [4,5] as the patients feel more involved and responsible for their own care [12]. In addition, patient portals might increase the patients’ satisfaction with care [13-18] and improve patient safety [15,17]. However, high-quality evidence of health benefits has not yet been demonstrated [8].

The potential benefits of patient portals cannot be utilized unless the patients accept and adopt the service [7,19]. According to the information system (IS) success model, the benefits of using the service arise from its use and user satisfaction [20]. Further, increased user satisfaction will lead to increased use [20], and unmet expectations will alter the use and satisfaction with patient portals [21]. Not all the barriers related to the use of patient portals are related to practical issues, such as a lack of hardware and access to the internet, but patients may have other valid reasons for nonuse as well [7,22,23]. Patients are also in an unequal position in terms of using electronic health care services, since not everyone has the resources or the same possibilities to use the services and take more responsibility for the management of their own health and well-being [24].

Previous research has examined differences in patient portal use in different contexts and patient populations. Several studies have reported an association between portal use and sociodemographic background [6,7,14,23,25,26] and various health-related factors [7,12,14,25,27,28]. In addition, patients’ guidance through increasing awareness and knowledge of patient portals [6,25,28], as well as endorsement and engagement with portals by health care professionals [7,25], have been identified as important associated factors. There are also some studies that have reported an association between the use of patient portals and factors related to the use of the internet, such as the frequency of use [26,29,30] and perceptions of the users’ own internet skills [26,30]. Furthermore, it has been reported that perceptions of electronic services may encourage or impede their use [31]. However, these previous studies have been conducted under normal circumstances before the COVID-19 pandemic and are thus only partially applicable in the context changed by the pandemic [4].

Factors associated with the patients’ satisfaction with patient portals have been less studied. Mainly descriptive research on the portal users’ experiences exists, and only little research has been conducted with quantitative methods about factors associated with satisfaction [5]. Kong et al [5] examined factors that predicted portal use and the users’ willingness to recommend the service among chronically ill patients during the COVID-19 pandemic in the Netherlands. They discovered that the respondent’s level of control, hospital visit time, life satisfaction, and level of depression are significantly associated with portal use. Variables related to the portal user’s waiting times for responses via the portal were the strongest predictors of the willingness to recommend the portal. However, the used variables concerned patients with long-term illnesses, and no comparisons were made to assess whether the same variables were associated with the use and willingness to recommend the patient portal. In addition, only little research exists on the factors associated with use and satisfaction using a nationally representative sample.

The aim of this study is to examine factors associated with the nonuse of and dissatisfaction with the Finnish nationwide patient portal My Kanta Pages (My Kanta) during the COVID-19 pandemic. Only respondents who had used the internet in the past 12 months were included to examine nonuse beyond the first-level digital divide [32] caused by a lack of hardware and access to the internet. Several factors related to (1) sociodemographic characteristics, (2) health and the use of health care services, (3) experiences of guidance concerning electronic services, and (4) digital skills and attitudes were examined. The evaluation of factors associated with the nonuse of and dissatisfaction with the patient portal is important to further develop the service and advocate for nonusers. Disparities in the use of patient portals might exacerbate the already existing disparities in health care access and health outcomes [33], increasing digital inequality in societies [34]. Knowledge of the factors that are associated with the nonuse of and dissatisfaction with the national patient portal in Finland, one of the pioneer countries in digitalization, can provide valuable information for countries and organizations that are further developing their electronic services.

## Methods

### Study Context

Finland is a sparsely populated country with 5.5 million residents. The health care system is decentralized, and until the end of 2022, municipalities (n=311) are responsible for organizing health care services, which are funded by taxes, state transfers, and user fees [1]. Finland can be considered 1 of the leading countries in terms of digitalization [35]. One of the most widely used electronic service is the nationwide patient portal called My Kanta. Between the years 2010 and 2018, cumulatively 63% of the adult Finnish residents had accessed the service. There is a professional user interface for Kanta Services, which can be used by the public and private actors of the social welfare and health care sector [36]. In addition to the nationwide patient portal, some public and private actors offer their clients access to their own patient portals or regional portals [37].

My Kanta was launched step-by-step starting from 2010 [38] to promote patient safety as well as the continuity and transparency of care [39]. My Kanta enables continuous access of Finnish residents to their health information [9], including browsing their own electronic prescriptions and medical records, such as patient reports, laboratory results, and X-ray statements [40]. All producers of health care services have been obligated to use electronic prescriptions since 2017 [41], and patients can request prescription renewals via My Kanta [42]. In addition, it is possible to record and monitor well-being data, such as blood glucose or activity meter. To access My Kanta, a Finnish personal identity code is needed, and e-authorization must be made with identification using online banking codes, mobile identification, or a certificate card [42].

The number of My Kanta users has grown steadily since its launch [38], and the COVID-19 pandemic increased the number of logins because of the availability of coronavirus test results [43]. In 2020, the service was used 29.4 million times by a total of 2.7 million individual visitors [43]. E-prescriptions were issued approximately 26.4 million times during 2020 [44]. In the future, the use is expected to further increase as the deployment of authorization for an adult to act on behalf of another adult was introduced after the data collection period and authorization for guardians to act, with some restrictions, on behalf of their children aged 10-17 years [45] will be fully implemented.

### Sample

This study was conducted in Finland as part of the FinSote 2020 National Survey of Health, Wellbeing, and Service Use [46]. The questionnaire was sent using stratified sampling to 13,200 Finnish residents who had reached the age of 20 years. Data were collected from September 2020 to February 2021 during the second wave of the COVID-19 pandemic. A possibility to respond either in electronic or in paper form in Finnish, Swedish, Russian, or English was offered. During the data collection, participants who had not responded were approached by mail up to 4 times.

Altogether, 6034 Finnish residents (n=3401 [56.4%] female, mean age 64.5 years, SD 17.9) responded to the questionnaire (response rate 46.5%). In total, 3919 (65.0%) respondents were included in the study sample as they had used health care services and the internet in the past 12 months. The sample was weighted using inverse probability weighting (IPW) correction [47]. The weights were estimated using sociodemographic register–based variables: the respondents’ age, gender, marital status, level of education, area of residence, and native language. Information about the respondents’ age, gender, and area of residence were obtained from the National Population Register. In previous research, the IPW method improved the accuracy of the results of a population survey and removed most of the bias caused by nonresponse in the various subpopulations [48].

### Ethics Approval

Participation in the study was completely voluntary. Ethical approval was obtained from the Ethics Committee of the Finnish Institute for Health and Welfare (THL/637/6.02.01/2017).

### Measurements

#### Dependent Variables

The *nonuse of My Kanta* was evaluated with the question “Have you used My Kanta in the past 12 months?” Respondents were asked to respond (1) *no* or (2) *yes*. For the analyses, the measure was binary-coded (0=user, 1=nonuser), and the users of My Kanta were set as the reference group.

The *dissatisfaction with My Kanta* was evaluated with a question concerning satisfaction: “If you have used the service, assess the quality of the service using a school grade (4-10).” In the Finnish education system, grades 8-10 represent grades from good to excellent and grades 4-7 from fail to satisfactory [49]. For the analyses, the measure was binary-coded (0=respondent was satisfied [grades 8-10] and 1=respondent was dissatisfied [grades 4-7] with the service), and the satisfied users of My Kanta were set as the reference group. Because the research interest was in respondents who were less satisfied with the service, respondents who gave an assessment of grade 7 were included in the group of dissatisfied users. This decision was also made based on substantive judgment to even the distribution [50] between satisfied and dissatisfied users, since only a small number of respondents had selected a grade from 4 to 6.

#### Independent Variables

Independent variables included characteristics concerning (1) sociodemographic background, (2) health and the use of health care services, (3) experiences of guidance concerning electronic services, and (4) digital skills and attitudes. All the used variables are presented in Multimedia Appendix 1.

#### Sociodemographic Characteristics

The respondents’ sociodemographic characteristics included their age, gender, education, and degree of urbanization. Age was used as a categorical variable in the descriptive statistics and as a continuous variable in all analyses. The *degree of urbanization* was determined according to the municipal classification and divided into 3 categories according to the proportion of people living in urban settlements and the population of the largest urban settlement: urban, semiurban, and rural municipalities [51]. Because of age-related differences in education, the respondents’ *educational level* was first divided into 10-year age groups by gender. Each group was divided into 3 categories based on their years of education, with approximately one-third of the respondents in each category: low, median, and high. Hence, the education-level variable had hardly any interaction with age and gender.

#### Health and the Use of Health Care Services

Variables concerning the respondents’ health and the use of health care services included self-rated health, long-term illness, and the use of health care services. *Self-rated health* was evaluated with a widely used, single-item measure of self-perceived health status. A subjective assessment of own health has been reported as a more sensitive measure in health monitoring than external measures of health, since it includes biological, psychological, and social dimensions. [52]. A scale from good to poor was used to evaluate the present state of the respondents’ health. In the analyses, the options (1) *good* and (2) *fairly good* were combined to represent good health, and the remaining options represented average or poor health. *Long-term illness* was binary-coded as (1) *yes* and (2) *no*. The *use of health care services* was binary-coded according to the number of annual outpatient appointments with a physician; 8 or more annual appointments were considered a high use of health care services, and less than 8 were counted as low or average use [53].

#### Experiences of Guidance Concerning Electronic Services

Variables concerning the experiences of guidance concerning electronic services included referrals to electronic services and the need for guidance on how to use online social or health care services. The *referral to electronic services* was evaluated with the question “If you have used social or health care services in the traditional way (paper, visit, or call) in the past 12 months, were you referred to electronic services (eg, My Kanta)?” For the analyses, option (1) *yes, I was referred* represented the respondents who were referred to electronic health care services. Option (2) was for those who were not referred to electronic health care services.

The *need for guidance* on using online social and health care services was evaluated with the statement “I need help with using online social and health care services.” In the analyses, the options (1) *completely agree* and (2) *somewhat agree* were combined as (1) *yes* and the remaining options as (2) *no* or *no opinion*.

#### Variables Related to Digital Skills and Attitudes

Variables related to digital skills and attitudes included digital skills, perceived benefits of electronic social and health care services, and security concerns. *Digital skills* were evaluated with 6 validated statements [54]. Based on pilot testing, 2 (33%) of the statements were transferred into positive statements [37]. A 5-point Likert scale was used to answer the statements (1=completely agree to 5=strongly disagree). Cronbach α for the statements was .86. In the analyses, a mean variable ranging from 1 to 5 was calculated for each respondent, and the measure was binary-coded to indicate (1) *good skills* (mean≤2.5) and (2) *poor skills* (mean>2.6). The same coding has previously been used in national research [37].

The *perceived benefits* of electronic social and health care services were measured with 8 statements. A 5-point Likert scale was used to answer the statements (1=completely agree to 5=strongly disagree). Cronbach α for the statements was .91. In the analyses, missing values were coded as *neither agree nor disagree*. A mean variable from 1 to 5 was calculated for each respondent, and the measure was binary-coded as (1) *beneficial* (mean≤2.5) and (2) *unbeneficial* (mean>2.6). The same coding has previously been used in national research [37].

*Security concerns* were evaluated with the statement “I am concerned about information security when it comes to my personal details”. In the analyses, options (1) *completely agree* and (2) *somewhat agree* were combined as (1) *yes* and the remaining options as (2) *no*.

### Statistical Analysis

In all statistical analyses, the IPW method [47] was applied to correct for bias by handling both differential sampling probabilities and missing data. Due to nonresponse in some items, the number of observations varied in the analyses.

Bivariate logistic regression analyses were used to examine the adjusted associations of respondent characteristics with the nonuse of My Kanta and dissatisfaction with the service (in separate analyses). First, univariate analyses, adjusted for age, gender, and education, were conducted at a time to examine the association of the dependent variable with each independent variable. Second, a multivariable model was formed, including only those independent variables with a *P* value of <.10. This cut-off for the *P* value was used for including the variables in the multivariable model, because the purpose was to identify potential independent variables rather than to test a hypothesis [55]. In the fully adjusted multivariable model, a *P* value of <.05 was considered statistically significant. Statistical methods suitable for weighted data were used, and SPSS Statistics version 27 was applied for the analyses.

## Results

### Characteristics

The weighted majority (3330/3919, 85.0%) of the respondents had used My Kanta in the past 12 months. Most of the My Kanta users (2841/3330, 85.3%) were satisfied with the service. A minority of respondents (589/3919, 15%) had not used My Kanta in the past 12 months.

The IPW weighted characteristics of the respondents representative of the Finnish population are presented in Tables 1-4. Almost half of the respondents were aged between 35 and 59 years. Over half (n=3401, 56.4%) of the respondents were female, and the majority lived in urban regions. Over half of the respondents were not referred to electronic health care services, such as My Kanta, by a health care professional. About half of the respondents perceived electronic health care services to be beneficial. The respondents who had used My Kanta in the past 12 months were mostly satisfied with the service (mean 8.31, SE .03), whereas a minority of users (489/3330, 14.7%) were dissatisfied. Over one-third (1359/3919, 34.7%) of the respondents had also used an electronic service provided by their occupational health care provider. Of these respondents, a minority (130/1359, 9.6%) only used the service provided by their occupational health care provider and not the nationwide patient portal My Kanta.

**Table 1:** Sociodemographic characteristics of the weighted study sample^a^.

Characteristics			Respondents, n (%)		Nonusers (N=589), n (%)		Dissatisfied users (N=489), n (%)
**Age, years (N=3919)**							
	20-34	918 (23.4)		144 (24.5)		120 (24.5)	
	35-59	1773 (45.2)		286 (48.5)		234 (48.0)	
	60-74	1008 (25.7)		129 (21.9)		107 (21.8)	
	75-99	220 (5.6)		30 (5.1)		28 (5.7)	
**Gender (N=3919)**							
	Male	1641 (41.9)		301 (51.1)		221 (45.2)	
	Female	2278 (58.1)		288 (48.9)		268 (54.8)	
**Education (N=3873)**							
	Low	1499 (38.7)		232 (39.3)		196 (40.1)	
	Median	1254 (32.4)		187 (31.8)		126 (25.8)	
	High	1120 (28.9)		170 (28.9)		167 (34.1)	
**Degree of urbanization (N=3919)**							
	Urban	2918 (74.5)		427 (72.5)		373 (76.4)	
	Semiurban	536 (13.7)		78 (13.3)		64 (13.1)	
	Rural	465 (11.9)		84 (14.2)		52 (10.6)	

^a^Inverse probability weighting (IPW)-corrected.

**Table 2:** Health and service use by the weighted study sample^a^.

Characteristics		Respondents, n (%)	Nonusers (N=589), n (%)	Dissatisfied users (N=489), n (%)
**Self-rated health (N=3893)**				
	Average or poor	1251 (32.1)	133 (22.6)	245 (50.2)
	Good	2642 (67.9)	456 (77.4)	244 (49.8)
**Long-term illness (N=3857)**				
	Yes	2165 (56.1)	191 (32.4)	319 (65.2)
	No	1692 (43.9)	398 (67.6)	170 (34.8)
**Use of health care services (N=3835)**				
	Low or average	3516 (91.7)	580 (98.5)	435 (89.0)
	High	319 (8.3)	9 (1.5)	54 (11.0)

^a^Inverse probability weighting (IPW)-corrected.

**Table 3:** Experiences of guidance concerning electronic services for the weighted study sample^a^.

Characteristics		Respondents, n (%)	Nonusers (N=589), n (%)	Dissatisfied users (N=489), n (%)
**Referral to electronic services (N=3166)**				
	Yes	1386 (43.8)	154 (26.2)	216 (44.1)
	No	1780 (56.2)	435 (73.8)	273 (55.9)
**Need for guidance (N=3833)**				
	Yes	379 (9.9)	86 (14.6)	74 (15.1)
	No	3454 (90.1)	503 (85.4)	415 (84.9)

^a^Inverse probability weighting (IPW)-corrected.

**Table 4:** Variables related to digital skills and attitudes of the weighted study sample^a^.

Characteristics		Respondents, n (%)	Nonusers (N=589), n (%)	Dissatisfied users (N=489), n (%)
**Digital skills (N=3897)**				
	Poor	199 (5.1)	48 (8.2)	25 (5.1)
	Good	3698 (94.9)	541 (91.8)	464 (94.9)
**Perceived benefits (N=3919)**				
	Yes	1840 (47.0)	252 (42.8)	181 (37.1)
	No	2079 (53.0)	337 (57.2)	308 (62.9)
**Security concerns (N=3823)**				
	Yes or N/A^b^	1923 (50.3)	323 (54.9)	312 (63.9)
	No	1900 (49.7)	266 (45.1)	177 (36.1)

^a^Inverse probability weighting (IPW)-corrected.

^b^N/A: not applicable.

### Associations With the Nonuse of My Kanta

Based on the results of age-, gender-, and education-adjusted univariate logistic regression analysis (Table 5), the following factors were included in the multivariable model: self-rated health, long-term illness, use of health care services, referral to electronic services, need for guidance, and digital skills.

The results of the fully adjusted logistic regression analysis regarding the nonuse of My Kanta are presented in Table 6. Male respondents were more likely to be nonusers of My Kanta compared to females. Respondents who used health care services to a low or average degree and who did not have a long-term illness were more likely to be nonusers of My Kanta compared to those who used health care services to a high degree and had a long-term illness. In addition, respondents who were not referred to electronic services, needed guidance, or had poor digital skills were over 2 times more likely to be nonusers of My Kanta compared to their counterparts.

**Table 5:** Results of univariate logistic regression analyses for the nonuse of My Kanta^a,b^.

Univariate analyses characteristics^c^		OR^d^ (95% CI)		*P* value^e^
**Sociodemographic characteristics**				
	Age (years)	0.84 (0.61-1.16)	.17	
	Gender (male)	1.61 (1.21-2.12)	.001	
	Low educational level	1.03 (0.73-1.46)	.85	
	Median educational level	1.01 (0.71-1.42)	.99	
	High educational level	Reference	N/A^f^	
**Degree of urbanization**				
	Urban	Reference	N/A	
	Semiurban	1.03 (0.70-1.52)	.87	
	Rural	1.34 (0.93-1.92)	.12	
**Health and service use**				
	Self-rated health (good)	1.75 (1.25-2.45)	.001	
	Long-term illness (no)	3.24 (2.41-4.36)	<.001	
	Use of health care services (low or average)	6.75 (2.49-18.31)	<.001	
**Experiences of guidance concerning electronic services**				
	Referral to electronic services (no)	2.43 (1.70-3.49)	<.001	
	Need for guidance (yes)	2.09 (1.44-3.05)	<.001	
**Variables related to digital skills and attitudes**				
	Digital skills (poor)	2.37 (1.50-3.76)	<.001	
	Perceived benefits (no)	1.25 (0.94-1.67)	.13	
	Security concerns (yes)	1.25 (0.94-1.67)	.13	

^a^Inverse probability weighting (IPW)-corrected.

^b^The model included the main effect of each variable adjusted for age, gender, and education.

^c^Reference categories indicated in parentheses: gender: male vs female; self-rated health: good vs average or poor; long-term illness: no vs yes; use of health care services: low or average vs high; referral to electronic services: no vs yes; need for guidance: yes vs no; digital skills: poor vs good; perceived benefits: no vs yes; security concerns: yes vs no.

^d^OR: odds ratio.

^e^Significance level of *P*<.10.

^f^N/A: not applicable.

**Table 6:** Results of the fully adjusted logistic regression analysis for the nonuse of My Kanta (N=2328)^a^.

Multivariable model characteristics^b^			OR^c^ (95% CI)		*P* value^d^
**Gender**					
	Female	Reference		N/A^e^	
	Male	1.67 (1.19–2.42)		.003	
**Self-rated health**					
	Average or poor	Reference		N/A	
	Good	1.48 (0.95–2.31)		.08	
**Long-term illness**					
	Yes	Reference		N/A	
	No	2.14 (1.48–3.10)		<.001	
**Use of health care services**					
	High	Reference		N/A	
	Low or average	4.66 (1.29–16.84)		.02	
**Referral to electronic services**					
	Yes	Reference		N/A	
	No	2.51 (1.70–3.71)		<.001	
**Digital skills**					
	Good	Reference		N/A	
	Poor	2.53 (1.32–4.83)		.01	
**Need for** **guidance**					
	No	Reference		N/A	
	Yes	2.26 (1.41–3.65)		<.001	

^a^Inverse probability weighting (IPW)-corrected.

^b^The model included all the independent variables with a *P* value of <.10 in the univariate model adjusted for age, gender, and education.

^c^OR: odds ratio.

^d^Significance level of *P*<.05.

^e^N/A: not applicable.

### Associations With Dissatisfaction With the Use of My Kanta

Based on the results of the age-, gender-, and education-adjusted univariate analyses (Table 7), the following variables were included in the multivariable model: education, self-rated health, long-term illness, need for guidance, perceived benefits, and security concerns. The results of the fully adjusted logistic regression analysis are presented in Table 8.

In the fully adjusted multivariable model, respondents who were younger, were male, and had a high level of education were more likely to be dissatisfied with My Kanta compared to their counterparts. Respondents with average or poor self-rated health were over 2 times more likely to be dissatisfied with My Kanta compared to respondents with a good perception of their own health. In addition, respondents who perceived electronic services as unbeneficial, who needed guidance, and who had security concerns were more likely to be dissatisfied with My Kanta compared to their counterparts.

**Table 7:** Results of univariate logistic regression analyses for dissatisfaction with My Kanta^a,b^.

Univariate analyses characteristics^c^		OR^d^ (95% CI)		*P* value^e^
**Sociodemographic characteristics**				
	Age (years)	0.99 (0.99–1.01)	.33	
	Gender (male)	1.31 (0.95–1.82)	.09	
	Low educational level	Reference	N/A^f^	
	Median educational level	0.72 (0.49–1.06)	.09	
	High educational level	1.11 (0.76–1.62)	.58	
**Degree of urbanization**				
	Rural	Reference	N/A	
	Semiurban	1.04 (0.60–1.79)	.89	
	Urban	1.08 (0.72–1.61)	.72	
**Health** **and service use**				
	Self-rated health (average or poor)	2.45 (1.79–3.36)	<.001	
	Long-term illness (yes)	1.34 (0.95–1.88)	.09	
	Use of health care services (high)	1.29 (0.76–2.21)	.35	
**Experiences of guidance concerning electronic services**				
	Referral to electronic services (no)	1.17 (0.82–1.67)	.38	
	Need for guidance (yes)	2.98 (1.83–4.85)	<.001	
**Variables related to digital skills and attitudes**				
	Digital skills (poor)	1.63 (0.88–3.03)	.12	
	Perceived benefits (no)	1.79 (1.30–2.47)	<.001	
	Security concerns (yes)	2.24 (1.61–3.13)	<.001	

^a^Inverse probability weighting (IPW)-corrected.

^b^The model included the main effect of each variable adjusted for age, gender, and education.

^c^Reference categories indicated in the parentheses: gender: male vs female; self-rated health: average or poor vs good; long-term illness: yes vs no; use of health care services: high vs average or low; referral to electronic services: no vs yes; need for guidance: yes vs no; digital skills: poor vs good; perceived benefits: no vs yes; security concerns: yes vs no.

^d^OR: odds ratio.

^e^Significance level of *P*<.10.

^f^N/A: not applicable.

**Table 8:** Results of the fully adjusted logistic regression analysis for dissatisfaction with My Kanta (N=2341)^a^.

Multivariable model characteristics^b^			OR^c^ (95% CI)		*P* value^d^
Age			0.99 (0.98–1.00)		.002
**Gender**					
	Female	Reference		N/A^e^	
	Male	1.40 (1.00–1.94)		.05	
**Education**					
	Low	Reference		N/A	
	High	1.48 1.02–2.16 ()		.04	
**Self-rated health**					
	Good	Reference		N/A	
	Average or poor	2.10 (1.51–2.93)		<.001	
**Long-term illness**					
	No	Reference		N/A	
	Yes	1.02 (0.71–1.45)		.92	
**Perceived benefits**					
	Yes	Reference		N/A	
	No	1.52 (1.09–2.11)		.01	
**Security concerns**					
	No	Reference		N/A	
	Yes	1.87 (1.33–2.62)		<.001	
**Need for** **guidance**					
	No	Reference		N/A	
	Yes	2.14 (1.33–3.46)		.002	

^a^Inverse probability weighting (IPW)-corrected.

^b^The model included all the independent variables with a *P* value of <.10 in the model adjusted for age, gender, and education.

^c^OR: odds ratio.

^d^Significance level of *P*<.05.

^e^N/A: not applicable.

## Discussion

### Principal Results

Most respondents of this nationally representative survey study had used the nationwide Finnish patient portal My Kanta in the previous 12 months and were satisfied with the service. However, more than every 10th user of health care services and the internet were nonusers of the national patient portal, and approximately the same number of users were dissatisfied with the service. Males and those in a need of guidance were more likely to be nonusers of the patient portal and dissatisfied with the service compared to women and those not needing guidance. Not having a long-term illness and low or average use of health care services were associated with the increased likelihood of nonuse of the My Kanta portal. In addition, respondents who were not referred to electronic services and who had poor digital skills were more likely to be nonusers of My Kanta compared to their counterparts. A younger age, higher education, and poor self-rated health were associated with an increased likelihood of dissatisfaction with the service. In addition, respondents who did not perceive electronic health care services to be beneficial and who had security concerns were more likely to be dissatisfied with the service compared to their counterparts.

### Strengths and Limitations

Finland is 1 of the forerunners of digitalization and ranked highest in information exchange and patient-centered information processing in an international comparative study [56]. By presenting the characteristics that are associated with nonuse of and dissatisfaction with the nationwide patient portal in Finland, valuable information can be offered for national initiatives for improvement and other countries aspiring to provide their residents with access to their health care documentation. However, generalizing our findings to countries with different levels of digitalization or service system should be done with caution.

A nationally presentative sample of Finnish residents was included in the analysis. The applied IPW method has previously been reported to improve the accuracy and generalizability of results [48]. However, the findings are based on self-reported data, with the possibility for bias, as respondents might not recall the previous happenings accurately. This could also lead to problems associated with common method variance and the inflation of the strength of relationships. In addition, some of the used independent variables are hard to explicitly measure and quantify because of their subjective nature, such as perceived benefits of electronic health care services. Although multiple factors were adjusted in the analyses, the possibility of residual confounding remains. Moreover, cross-sectional survey data do not allow drawing any confirmatory causal inferences from the results.

Since only respondents who had used the internet for transactions or for searching for information were included in the study, the results are only applicable when considering the nonuse of patient portals beyond the first-level digital divide caused by the lack of necessary devices and access to the internet. Some respondents who did not use My Kanta used additional patient portals provided by private or public providers of health care services. It is also noteworthy that some respondents might not be referred to electronic health care services, because their transactions in health care do not require further action or electronic services cannot provide support in their situation. The research concerning the users and nonusers of nationwide patient portals is sparse, and comparison is difficult as the properties provided in the portals vary, in addition to the differing patient populations and adjustments in the analyses.

### Comparison With Prior Work

The use of nationwide patient portals varies by country and portal [9,10]. This study showed that the majority of Finnish residents who had used health care services and the internet in the past 12 months had used the nationwide My Kanta patient portal. The use of the patient portal increased during the COVID-19 pandemic because of the availability of coronavirus test results, easing the burden on health care services [43]. The availability of the test results has likely increased the use of My Kanta among those with no long-term illness and low use of health care services. This may also suggest that a larger proportion of this group among the respondents was reached for this study than would have been reached before the pandemic. Approximately only every 10th user of the patient portal was dissatisfied, indicating a high level of satisfaction with the service. It might be presumed that the restrictions for avoiding face-to-face encounters and fear of the infection increased the overall satisfaction with electronic health care services during the COVID-19 pandemic. However, even before the pandemic, high satisfaction with My Kanta has been reported [57-59].

The results of this study suggested that younger and more educated respondents were more likely to be dissatisfied with My Kanta compared to older and less educated respondents. The findings of this study were supported by the fact that younger generations have grown up with technology and were thus more comfortable using electronic services [60], which might result in higher expectations toward the services. Previous research on a Norwegian symptom checker [61] reported that compared to younger users, older users were more satisfied with the service because they tended to navigate it in a more superficial way without gaining awareness of the existing problems. Contradictory to the findings of this study, patients with higher education have been reported to be more satisfied with telemedicine compared to patients with lower education [62]. However, only participants with moderate or high levels of digital health literacy were recruited, which might explain the contradictory results. Among the less educated respondents, digital health literacy skills might be an important factor leading to dissatisfaction with the service [63].

This study found that respondents without any long-term illness and with low or average use of health care services are more likely to be nonusers of My Kanta, which is consistent with previous research [7,19,25,27,64]. Patients without long-term illness and lower use of health care services might have fewer needs related to health care and the use of patient portals. In addition, good self-rated health was associated with patient portal nonuse in this study, similar to studies by Moll et al [27] and Zanaboni et al [12], but only before controlling for the use of health care services and long-term illness. However, even after these adjustments, a more negative perception of patients’ own health was associated with dissatisfaction with the service. Previous research has reported an almost linear association between the patients’ poor perception of their own health and the number of annual outpatient visits with a physician [52]. It can be anticipated that patients with poor self-rated health have more health care needs and fewer resources and, thus, presumably higher expectations of patient portals, which may lead to dissatisfaction.

Over half of the respondents were not referred to electronic health care services by their care providers, and the nonreferred respondents were less likely to use the nationwide patient portal My Kanta compared to referred respondents. Sääskilahti et al [29] and Kong et al [5] have also reported unfamiliarity with the service among nonusers of patient portals. It is important to highlight the role of professionals and health care managers in activating and engaging patients to accept and use patient portals, because promotion is heavily associated with their use [7,29,65-69]. The promotion should be integrated into routine care processes, and individual training and support should be provided on the portal use to patients with different background demographics to help prevent the digital divide from widening [25,67,69-71]. In addition to the promotion by professionals, alternative means are also needed to increase adoption [70]. Further research is necessary to identify effective ways to integrate patient portal enrollment into clinical practice. After data collection, My Kanta received national publicity because of active marketing of the EU Digital COVID Certificate, which can be downloaded from the portal. This has further increased the public’s awareness of My Kanta. In the future, it might be expected that increasingly more patients have prior knowledge and experience with the service. This might also represent a significant incentive for the public’s further adoption of electronic services [4].

Although patients have prior experience in the use of electronic services and information technology tools, the ability to review and manage medical records on patient portals should not be assumed [28]. Results of this study suggest that respondents who needed guidance in the use of electronic health care services were more likely to be nonusers of the portal or dissatisfied with the service compared to respondents without a need for guidance. It is likely that perception of a lack of ability in the use of electronic health care services or poorly designed services will alter the use and the benefits from their use, leading to dissatisfaction with the portal.

The previous literature has suggested different ways in which the use of patient portals could be promoted, including ease of entry [13,30,72], easy navigation [73,74], and reducing the required cognitive demands [73]. Electronic services should meet certain accessibility requirements [75,76] in order to support the equality of the users. Many providers of electronic health care services still struggle with meeting the demands of accessibility, impairing the position of especially patients with disabilities [77]. My Kanta fulfills these requirements at a certain level by assessing and reporting the current status of the accessibility and providing an electronic channel for feedback [78].

In addition to easy accessibility of the service, assistance on the use should be available at a low threshold. Because patients have previously been reported to seldom seek help from family members, friends, service support, or health care providers [12], electronic services should be designed to include assistance to users. New electronic introductory and teaching materials have been prepared by the system administrator of My Kanta [79], and health care professionals should guide their clients to these materials. Patients with different background demographics were involved to a limited extent in the development of these materials. Efforts to stimulate participation of especially disadvantaged patients in developmental work of patient portals [80] and educational materials should be highlighted.

Digital skills are necessary for wider patient adoption and use of patient portals [31,34,37,81,82]. The findings of this study are similar to previous research [26,30] as respondents with poor digital skills were more likely to be nonusers of My Kanta portal compared to respondents with good skills. For the patients to be able to effectively navigate the portal, their digital competence needs to be promoted [71]. The Finnish national strategy for applying information technology to health care and social welfare currently states that Finnish residents should be able to use electronic services and produce self-recorded data to promote their well-being [83]. However, by making digital skills a policy priority, the equal use of electronic health care services and patient portals could be promoted and the risk for digital divide minimized [82]. Good digital skills have also been reported to be associated with the perception that electronic services are more useful [24].

Attitudes about the usefulness, appropriateness, and potential downsides of electronic services may encourage or impede the use [31]. In this study, the respondents who did not perceive overall benefits in electronic health care services were more likely dissatisfied with My Kanta compared to respondents with a more positive attitude. Negative attitudes have previously been reported to alter patient satisfaction with the patient portal [69]. To ensure the widespread and equal use of electronic health care services, all users must experience them as beneficial [24]. Patients’ perceptions of the benefits can be increased by offering them demonstrations and information about the capabilities of the patient portal [69,84]. Perceived benefits were not associated with the nonuse of the patient portal according to the results of this study.

According to this study, respondents who had security concerns were more likely to be dissatisfied with My Kanta compared to respondents who felt more secure. Similar to the results of Woods et al [30], security concerns were not associated with the use of the patient portal. Privacy, security, and confidentiality concerns regarding medical information have been identified as barriers to the use of patient portals, and some patients may feel discomfort at having their personal health information on the internet [25,66,69,85]. Attention needs to be paid to information security and identity protection, since these are critical issues and central to widespread consumer acceptance and adoption of patient portals [82]. Security concerns also complicate requests for assistance and guidance from non-health-care professionals as well as the use of patient portals on public computers, since others might be able to see sensitive medical information on the screen [69,84]. Private facilities should be promoted in libraries and other places with public computers. The COVID-19 pandemic has complicated the requests for assistance and the use of computers in public facilities because of societal restrictions. More research is needed to understand how safety concerns could be alleviated. In addition, technology users of all ages should be equipped with knowledge of online privacy and security as a new set of cyber security skills are needed in the increasingly digital society [86].

### Conclusion

According to the results of this population-based cross-sectional survey study in the era of COVID-19, patients without long-term illnesses, those not referred to electronic health care services, and those in need of guidance on the use of online social and health care services seem to be more likely nonusers of the Finnish nationwide patient portal My Kanta. Moreover, poor health and security concerns seem to be associated with dissatisfaction with the service. Interventions to promote referral to electronic health care services by professionals are needed. Attention must be paid to information security of the service as well as the alleviation of the patients’ privacy concerns.

## Appendix

Multimedia Appendix 1 Description of the used variables.

## Abbreviations

IPW: inverse probability weighting
OR: odds ratio
