# Supplementary material for: The Factors Associated With Nonuse of and Dissatisfaction With the National Patient Portal in Finland in the Era of COVID-19: Population-Based Cross-sectional Survey
Source: JMIR Med Inform. 2022 Apr 22;10(4):e37500. doi: 10.2196/37500 (PMC9037616; doi:10.2196/37500)
Supplement: Multimedia Appendix 1 [file medinform_v10i4e37500_app1.docx]

Multimedia Appendix 1.

### The used variables

*The non-use of and dissatisfaction with My Kanta*

Have you used any of the following electronic services in social or healthcare in the past 12 months? If you have used the service, access the quality of the service using a school grade (4-10).

**My Kanta Pages (e.g., personal prescriptions and health data)**

[_] no [_] yes school grade _______

*Self-rated health*

**How would you describe your state of health at present?**

[_] good [_] fairly good [_] average [_] fairly poor [_] poor

L*ong-term illness*

**Do you have any longstanding illness or health problem?**

[_] yes [_] no

*Use of healthcare services*

**How many times in the past 12 months have you seen a doctor in an appointment or at you home because of an illness you have had (or because of pregnancy or childbirth?** If you have not seen a doctor at all, please enter 0. This does not include any times when you have been admitted to a hospital as an inpatient.

at a health station (no dental appointments) _______ times

at a private medical clinic _______ times

in occupational healthcare _______ times

at a hospital outpatient clinic _______ times

at home _______ times

elsewhere (e.g., student healthcare service, military healthcare service) _______ times

*Referral to electronic services*

**If you have used social or health care services in the traditional way (paper, visit, or call) in the last 12 months, were you referred to electronic services (e.g., My Kanta Pages)?**

[_] yes, I was referred [_] no, I wasn’t referred [_] I haven’t used them [_] cannot say

*Need for guidance*

**How do you feel about the following claims about electronic services?**

**I need help with using online social and healthcare services**

[_] completely agree [_] somewhat agree [_] neither agree nor disagree [_] somewhat disagree [_] strongly disagree

*Digital skills*

**Assess your ability to use the internet**

**I know how to open the webpage I want**

[_] completely agree [_] somewhat agree [_] neither agree nor disagree [_] somewhat disagree [_] strongly disagree

**I know how to connect to a WIFI network**

[_] completely agree [_] somewhat agree [_] neither agree nor disagree [_] somewhat disagree [_] strongly disagree

**It is easy for me to choose the right search terms when I am looking for information online**

[_] completely agree [_] somewhat agree [_] neither agree nor disagree [_] somewhat disagree [_] strongly disagree

**I can assess the reliability of online information**

[_] completely agree [_] somewhat agree [_] neither agree nor disagree [_] somewhat disagree [_] strongly disagree

**I know how to complete online forms (e.g. tax return, passport application)**

[_] completely agree [_] somewhat agree [_] neither agree nor disagree [_] somewhat disagree [_] strongly disagree

**I know how to download apps to my mobile device**

[_] completely agree [_] somewhat agree [_] neither agree nor disagree [_] somewhat disagree [_] strongly disagree

*Perceived benefits*

**How do you feel about the following claims concerning the benefits of electronic social and healthcare services?** If you cannot assess the electronic services, choose “neither agree nor disagree”.

**Help me to maintain healthy lifestyle**

[_] completely agree [_] somewhat agree [_] neither agree nor disagree [_] somewhat disagree [_] strongly disagree

**Help me to assess the need for services**

[_] completely agree [_] somewhat agree [_] neither agree nor disagree [_] somewhat disagree [_] strongly disagree

**Supports me in finding and choosing the most suitable service**

[_] completely agree [_] somewhat agree [_] neither agree nor disagree [_] somewhat disagree [_] strongly disagree

**Make it easier for me to use services regardless of where I am and when**

[_] completely agree [_] somewhat agree [_] neither agree nor disagree [_] somewhat disagree [_] strongly disagree

**Make it easier for me to collaborate with professionals**

[_] completely agree [_] somewhat agree [_] neither agree nor disagree [_] somewhat disagree [_] strongly disagree

**Help me to take an active role in looking after my own health and welfare**

[_] completely agree [_] somewhat agree [_] neither agree nor disagree [_] somewhat disagree [_] strongly disagree

**Help tailor the service to my individual needs**

[_] completely agree [_] somewhat agree [_] neither agree nor disagree [_] somewhat disagree [_] strongly disagree

**Help me to take care of the health, welfare and functional capacity of family or friends**

[_] completely agree [_] somewhat agree [_] neither agree nor disagree [_] somewhat disagree [_] strongly disagree

*Security concerns*

**How do you feel about the following claims about electronic services?**

**I am concerned about the information security when it comes to my personal details**

[_] completely agree [_] somewhat agree [_] neither agree nor disagree [_] somewhat disagree [_] strongly disagree
